# Clinical Events with Edoxaban in South Korean and Taiwanese Atrial Fibrillation Patients in Routine Clinical Practice

**DOI:** 10.3390/jcm10225337

**Published:** 2021-11-16

**Authors:** Eue-Keun Choi, Wei-Shiang Lin, Gyo-Seung Hwang, Paulus Kirchhof, Raffaele De Caterina, Cathy Chen, Martin Unverdorben, Chun-Chieh Wang, Young-Hoon Kim

**Affiliations:** 1 Department of Internal Medicine, Seoul National University College of Medicine, Seoul National University Hospital, Seoul 03080, Korea; 2 Division of Cardiology, Tri-Service General Hospital, National Defense Medical Center, Taipei 114, Taiwan; wslin545@ms27.hinet.net; 3 Department of Cardiology, Ajou University Hospital, Suwon 442-721, Korea; hwanggs@medimail.co.kr; 4 Institute of Cardiovascular Sciences, University of Birmingham and SWBH, UHB NHS Trusts, IBR 136, Wolfson Drive, Birmingham B15 2TT, UK; p.kirchhof@uke.de; 5 Department of Cardiology, University Heart and Vascular Center Hamburg, Martinistraße 52, 20251 Hamburg, Germany; 6 German Center for Cardiovascular Research (DZHK) Partner Site Hamburg/Kiel/Lübeck, 23562 Lübeck, Germany; 7 Department of Cardiology, University of Pisa, 56126 Pisa, Italy; raffaele.decaterina@unipi.it; 8 Fondazione Villa-Serena per la Ricerca, Città Sant’Angelo, 65013 Pescara, Italy; 9 Global Specialty Medical Affairs, Daiichi Sankyo, Inc., Basking Ridge, NJ 07920, USA; cchen@dsi.com (C.C.); munverdorben@dsi.com (M.U.); 10 Department of Cardiology, Chang Gung Memorial Hospital, Chang Gung University, Taoyuan 333, Taiwan; chcwang@cgmh.org.tw; 11 Department of Internal Medicine, Korea University College of Medicine, Korea University Medical Center, Seoul 02841, Korea; yhkmd@unitel.co.kr

**Keywords:** atrial fibrillation, edoxaban, real-world data, Asia, ETNA

## Abstract

Edoxaban is approved for stroke prevention in nonvalvular atrial fibrillation (AF) patients in numerous countries. Outcome data are sparse on edoxaban treatment in AF patients from routine clinical practice, especially in Asian patients. Global ETNA (Edoxaban in rouTine cliNical prActice) is a noninterventional study that integrates data from patients from multiple regional registries into one database. Here, we report the 1-year clinical events from AF patients receiving edoxaban in South Korea and Taiwan. Clinical events assessed included bleeding, strokes, systemic embolic events, transient ischemic attacks (TIAs), and all-cause and cardiovascular death. Overall, 2677 patients (mean (range) age 72 (66–78) years, male 59.7%, mean CHA_2_DS_2_-VASc score ± standard deviation 3.1 ± 1.4) were treated with 60 or 30 mg edoxaban and had 1-year follow-up data. The annualized event rates for major bleeding and clinically relevant non-major (CRNM) bleeding were 0.78% and 0.47%, respectively. Annualized event rates for ischemic stroke and hemorrhagic stroke were 0.90% and 0.19%, respectively. Event rates for major and CRNM bleeding and rates of ischemic stroke and TIA were higher in Taiwanese patients than in Korean patients. Event rates were low and similar to those found in other studies of edoxaban in Korean and Taiwanese AF patients, thus supporting the safety and effectiveness of edoxaban in this population.

## 1. Introduction

Oral anticoagulants—including vitamin K antagonists (VKAs) and direct oral anticoagulants (DOACs) such as dabigatran, rivaroxaban, apixaban, and edoxaban—are effective in the prevention of stroke in patients with atrial fibrillation (AF) and for the treatment and prevention of venous thromboembolism (VTE) [1,2,3,4]. The Effective Anticoagulation with Factor Xa Next Generation in Atrial Fibrillation–Thrombolysis in Myocardial Infarction 48 (ENGAGE AF-TIMI 48) randomized controlled clinical trial showed that compared with well-managed warfarin, once-daily edoxaban showed a similar efficacy for stroke or systemic embolic event (SEE) prevention, with lower rates of bleeding and cardiovascular death [5]. Based on these data, edoxaban is approved in numerous countries, including South Korea (hereafter, Korea) and Taiwan, for the prevention of stroke and SEE in patients with nonvalvular AF.

The prevalence of AF has increased in both Korea and Taiwan over the last two decades [6,7]. In Taiwan, the frequency of diagnosed AF demonstrated a 1.65-fold increase between 1997 and 2002, and the incidence of AF in Korea increased 1.7-fold from 2008 to 2015 [6,7]. With the increased prevalence of AF in these two Asian nations, it is important to understand how the condition is being managed in routine clinical practice. However, there is limited data on clinical events with DOAC treatment in patients with AF from clinical settings—especially in Asian patients.

Noninterventional studies provide important information that complements randomized controlled studies and are beneficial in documenting patient safety and effectiveness in routine clinical practice [8,9,10]. To that end, Global ETNA (Edoxaban in rouTine cliNical prActice) is a noninterventional study designed to integrate data from patients receiving edoxaban from multiple regional registries into a single, global database [11,12,13]. It is the largest single drug DOAC noninterventional study conducted to date. One-year clinical event rates from 26,823 AF patients enrolled in the Global ETNA program from Europe and Asia demonstrated low rates for stroke and major bleeding, with notable differences in patient demographics and clinical events across regions [13]. Here, we report the results of an analysis of 1-year clinical events in AF patients receiving edoxaban in Korea and Taiwan.

## 2. Methods

### 2.1. Study Design

The overall design of the Global ETNA program has previously been described [12]. The Global ETNA program integrates prospective, observational noninterventional regional studies from Europe (Germany, Austria, Switzerland, Belgium, Italy, Spain, UK, Ireland, the Netherlands, and Portugal), Japan, Korea, and Taiwan, as well as other East and Southeast Asian countries. The program includes patients with AF prescribed edoxaban for stroke prevention (ETNA-AF) and patients receiving edoxaban for the treatment and secondary prevention of venous thromboembolism (ETNA-VTE). The current analysis focuses on 1-year clinical events in patients with AF from Korea and Taiwan enrolled in the ETNA-AF program.

The ETNA protocols were approved in all participating countries by the responsible ethics committees and institutional review boards prior to initiation. All ETNA studies were conducted in accordance with national laws and regulations. Korea and Taiwan adhered to the Guidelines for Good Pharmacoepidemiology Practice and the European Medicines Agency (EMA) Guidelines in Good Pharmacovigilance Practices Module VIII (management and reporting of adverse reactions to medicinal products; EMA/813938/2011 rev 1).

### 2.2. Patient Selection

Eligible AF patients were those treated with edoxaban for stroke prevention according to the local label. This includes patients on the standard once-daily 60 mg dose and patients who were reduced to the 30 mg dose for meeting any one of the three label-indicated dose-reduction criteria (renal impairment (creatinine clearance: 15–50 mL/min), weight ≤ 60 kg, or for concomitant use of certain P-glycoprotein inhibitors) [14]. The only other exclusion criteria were not providing written informed consent or participating in a simultaneous interventional study.

### 2.3. Assessment and Clinical Events

Baseline information collected included demographics, vital signs, AF history and diagnosis, renal and hepatic function parameters, bleeding history, and the specifics of edoxaban therapy. A patient was considered as having a medical history of heart failure (HF) if one of the following criteria was fulfilled: documented congestive heart failure (CHF) or, if CHF was not documented, then documentation of ischemic cardiomyopathy; ejection fraction <40%; frequent dyspnea (≥1/day) without chronic obstructive pulmonary disease and with documented severe valvular heart disease, coronary heart disease post-myocardial infarction (MI), valve replacement, or hypertension treated with ≥3 drugs.

Clinically relevant events assessed included first or recurrent ischemic stroke, hemorrhagic stroke, SEE, bleeding, transient ischemic attack (TIA), MI, acute coronary syndrome, all-cause death, and cardiovascular (CV)-related death. Bleeding was characterized as major, clinically relevant non-major (CRNM), or minor in accordance with the International Society on Thrombosis and Haemostasis [15].

### 2.4. Documentation and Data Assessment

Data were collected at baseline and at 12 and 24 months after enrollment. All ETNA-AF data were provided by treating physicians and obtained from medical records. Electronic case report forms and predefined definitions and variables were used. Free text field documentation was limited. Medical history, clinical events, and adverse drug reactions were coded using the Medical Dictionary for Regulatory Activities. Clinical events were based on physician diagnosis and assessment. The event analysis was based on dates with imputation.

### 2.5. Statistical Analyses

All data analyses are exploratory and descriptive in nature. Results are presented by summary statistics. Baseline data are presented as frequencies and/or with summary statistics. The number of patients with at least one clinical event is presented (*n* (%)) separately for each type of clinical event. Only events with a start date greater than the date of baseline and less than the date of baseline +365 days were included.

## 3. Results

### 3.1. Baseline Demographics and Clinical Characteristics

At 1-year follow up, 2713 unselected patients with AF from 47 centers who were taking edoxaban for stroke prevention were enrolled in this registry, and 2677 were treated with either 60 mg or 30 mg edoxaban. Of these, 48.7% were treated with 60 mg edoxaban, and 51.3% were treated with 30 mg edoxaban (Table 1). Overall, 1134 patients (42.4%) met at least one criterion for dose reduction (renal impairment, body weight ≤60 kg, or concomitant use of certain P-glycoprotein inhibitors), and 76.1% of these patients were prescribed the correct 30 mg dose, whereas the other 23.9% were taking the non-recommended 60 mg. There was a higher percentage of patients from Taiwan taking the 30 mg dose (56.5% vs. 48.6%) compared with Korea; this may be explained by the lower median creatinine clearance (CrCl) in patients from Taiwan compared with those from Korea (median (interquartile range, IQR): 56.9 (41.7, 72.4) vs. 62.6 (49.5, 79.1)). Similar proportions of patients from Korea vs. Taiwan (29.0% vs. 29.5%) were receiving an off-label dose (60 mg for patients who met the dose-reduction criteria or 30 mg for patients that did not meet dose-reduction criteria).

**Figure 1:**
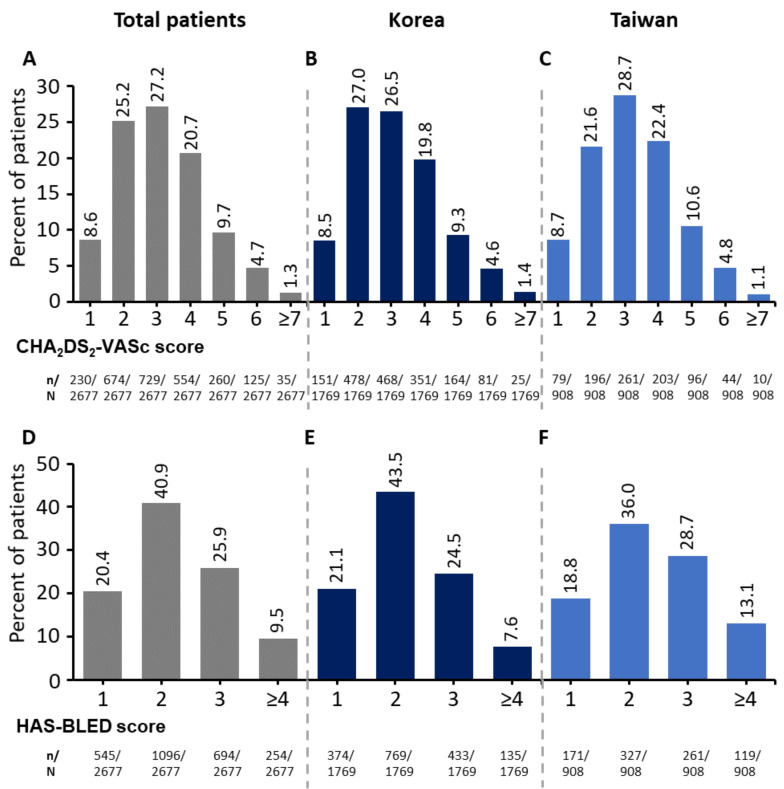
Distribution of CHA_2_DS_2_-VASc and HAS-BLED Scores. Distribution of CHA_2_DS_2_-VASc (**A**–**C**) and HAS-BLED (**D**–**F**) scores for total patients (**A**,**D**), patients from Korea (**B**,**E**) and patients from Taiwan (**C**,**F**). A total of 70 patients (2.6%) had a CHA_2_DS_2_-VASc score of 0; 88 patients (3.3%) had a HAS-BLED score of 0. CHA_2_DS_2_-VASc, congestive heart failure, hypertension, age, diabetes, prior stroke/TIA, vascular disease, and sex category; HAS-BLED, hypertension, abnormal renal and liver function, stroke, bleeding history or disposition, Labile INR, elderly, drugs or alcohol; INR, international normalized ratio; TIA, transient ischemic attack.

The median age was 72 years (IQR (66,78)) and 40.5% of patients were ≥75 (Table 1). Most patients (59.7%) were male. The baseline CHA_2_DS_2_-VASc score was 3.1 ± 1.4, and the HAS-BLED score was 2.2 ± 1.0 (mean ± standard deviation (SD)). Approximately 73% of patients had a CHA_2_DS_2_-VASc score of 2–4, and 16% of patients were at very high risk, with a score ≥5. A total of 67% of patients had a HAS-BLED score of 2 or 3, indicating a moderate-to-high risk of bleeding (Figure 1). Patients presented with multiple comorbidities including hypertension (71.3%), diabetes mellitus (29.4%), coronary artery disease (CAD; 15.4%), prior ischemic stroke (14.5%), and HF (12.1%; Table 1). Patients receiving the 30 mg dose, compared with those on 60 mg edoxaban, presented with more prognosis-relevant factors such as lower CrCl (mean (SD): 59.2 (24.6) vs. 73.3 (21.1)) and, in their medical histories, more major (2.8% vs. 1.3%) and CRNM bleeding events (1.2% vs. 0.5%).

The demographics were similar between patients from Korea and Taiwan; however, there were some notable differences. There was a higher percentage of patients aged ≥75 years from Taiwan (45.7%) compared with Korea (37.8%), and 61.6% of patients from Taiwan had paroxysmal AF, whereas, in Korea, the percentage was 34.4% (Table 1). In addition, there were more patients with histories of HF and CAD in Taiwan vs. Korea (16.2% vs. 10.0% and 20.0% vs. 13.0%). Conversely, more patients in Korea had a history of ischemic stroke vs. Taiwan (17.8% vs. 8.3%; Table 1).

### 3.2. Bleeding Events

**Figure 2:**
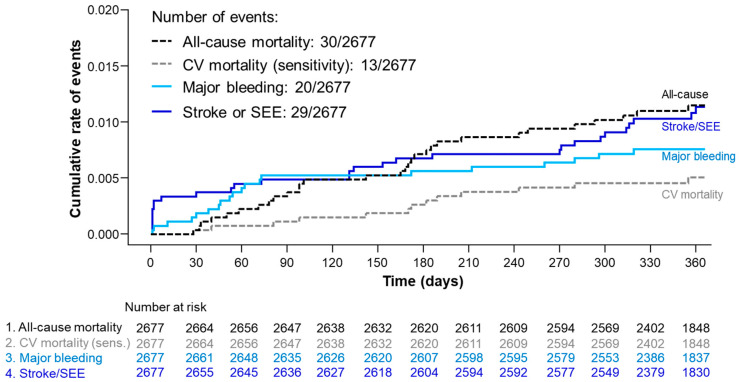
Kaplan-Meier curves for all-cause mortality, CV mortality, major bleeding, and stroke or SEE. CV, cardiovascular; SEE, systemic embolic event; sens, sensitivity.

The annualized event rates for major bleeding, intracranial hemorrhage (ICH), major gastrointestinal (GI) bleeding, and CRNM bleeding were 0.78%, 0.27%, 0.23%, and 0.47%, respectively (Table 2 and Figure 2). All bleeding event rates (major ICH, major GI bleeding, and CRNM bleeding) were higher in patients from Taiwan than in Korea (Table 2). Patients receiving the 30 mg dose of edoxaban had higher annualized event rates for major bleeding (1.31%) compared with patients receiving the 60 mg dose (0.24%); this was true for patients in both Korea and Taiwan (Supplementary Table S1). Overall, 1896 patients (70.8%) were prescribed an on-label dose of edoxaban, whereas 510 patients (19.1%) were underdosed with off-label 30 mg edoxaban. Off-label dosing did not appear to influence bleeding event rates (Supplementary Table S2).

### 3.3. Effectiveness

Overall, the annualized event rates for ischemic stroke, TIA, and hemorrhagic stroke were 0.90%, 0.19%, and 0.19% (Table 2 and Figure 2). Annualized event rates for MI and SEE were 0.16%, and 0.04% (Table 2). There was a total of 30 all-cause deaths, of which 13 were CV-related (annualized event rates of 1.17% and 0.51%, respectively; Table 2 and Figure 2). Compared with Korea, patients from Taiwan had numerically higher annualized rates of ischemic stroke (1.29% vs. 0.71%), MI (0.23% vs. 0.12%), TIA (0.47% vs. 0.06%), all-cause mortality (1.98% vs. 0.76%), and CV-related mortality (0.58% vs. 0.47%). Overall, patients receiving the 30 mg edoxaban dose had a similar annualized event rate for ischemic stroke (0.92%) as patients receiving the 60 mg edoxaban dose (0.88%). In Korea, patients receiving the 60 mg dose had a higher rate of ischemic stroke compared with patients receiving the 30 mg dose (0.92%/year vs. 0.48%). However, the opposite was observed in Taiwan: patients receiving the 60 mg dose had a lower rate of ischemic stroke (0.80%/year) than patients receiving the 30 mg dose (1.67%/year; Supplementary Table S1). The annualized rates of all-cause and CV-related mortality were higher for patients receiving the 30 mg edoxaban dose (1.83% and 0.76%, respectively) vs. the 60 mg edoxaban dose (0.48% and 0.24%, respectively), regardless of country of origin (Supplementary Table S1). Off-label dosing did not appear to influence effectiveness (Supplementary Table S2).

## 4. Discussion

This analysis from the Global ETNA-AF program included 2677 AF patients from Korea and Taiwan with 1-year follow-up data. The baseline patient demographics and medical histories reveal many differences between the two populations that may be relevant to the clinical event rates. For example, patients from Taiwan were slightly older (with a higher percentage of patients aged ≥75 years) with lower CrCl, and a higher percentage had a history of major GI bleeding, HF, CAD, peripheral artery disease, and chronic obstructive pulmonary disease compared with those from Korea. Additionally, a higher percentage of Taiwanese patients had a CHA_2_DS_2_-VASc score ≥4 compared with Korean patients (38.9% vs. 35.1%, respectively). Conversely, a higher proportion of Korean patients had a history of ischemic stroke. Taiwan also had a higher proportion of patients diagnosed with paroxysmal AF and a higher percentage of patients taking antiarrhythmic and/or rate control drugs. These data indicate that patients from Taiwan compared with Korea have an overall higher burden of comorbidities. One-year clinical events were low in Korean and Taiwanese patients; however, the higher burden of pre-existing diseases and advanced age in Taiwanese patients is evidenced in the consistently higher bleeding, thromboembolic clinical events, and all-cause mortality in this population.

The Global ETNA-AF study enrolled 26,823 patients with AF, with 48.8% from Europe, 41.2% from Japan, and 10.0% from South Korea and Taiwan [13]. At the 1-year follow up, the annualized event rates for major bleeding, ICH, and major GI bleeding were 1.12%, 0.31%, and 0.57%, respectively [13]. The annualized event rates of stroke, CV death, and all-cause death were 1.12%, 1.22%, and 3.03%, respectively [13]. Clinical event rates for the Global ETNA-AF population were notably larger than those for the South Korean and Taiwanese population presented in the current manuscript (Table 3). Regional differences in select clinical events were apparent in the Global ETNA study. For example, the rates of all-cause death and CV death in Europe were 3.50%/year and 1.63%/year, respectively, whereas these rates in Japan were 2.91%/year and 0.85%/year, respectively [13]. Although causal relationships have not yet been established, regional differences in patient demographics and baseline risk factors are likely a key contributor to the varying clinical event rates published for patients with AF treated with edoxaban at a 1-year follow up.

The overall major bleeding annualized event rate in this study was 0.78%. In the prespecified secondary analysis of 1943 East Asian patients (including patients from Japan, China, Taiwan, and Korea) from the ENGAGE AF-TIMI 48 trial, the major bleeding rate was 2.86% [16]. Without Japan in this analysis, the rate of major bleeding was 2.23% [17]. The disparity between ENGAGE AF-TIMI 48 and the current analysis from ETNA-AF may be explained by the higher stroke risk score in ENGAGE AF-TIMI 48 [5]. Overall, the reported incidence of ICH with warfarin was 1.5–2-fold higher in Asian vs. Caucasian patients [18,19]. It is of particular note that in the East Asian population in the ENGAGE AF-TIMI 48 trial, the event rate for ICH with the high-dose edoxaban regimen was 3-fold higher with VKA [16]. A study using the Korean health claims database found that Korean patients taking edoxaban (60/30 mg) had a similarly low incidence of ICH compared with patients taking warfarin (0.66 vs. 1.59 per 100 person-years, respectively) [18]. In the current analysis, the event rates for ICH were also low (0.27%). Data from both of these large registry studies are in line with the low rate of ICH observed with edoxaban treatment in an East Asian population in the ENGAGE AF-TIMI 48 trial.

The low rate of stroke and major and CRNM bleeding events observed in this ETNA-AF study are consistent with those reported in other real-world population-based studies. For instance, edoxaban was associated with a lower risk of ischemic stroke and hospitalization for GI bleeding or major bleeding than warfarin in the Korean National Health Insurance Service database. This study also reported a lower incidence of all-cause mortality in patients taking edoxaban compared with warfarin [18]. Although ETNA-AF did not include a comparator arm, the event rates with edoxaban reported here were similar to those reported with edoxaban in the previous Korean health claims database. Similarly, a retrospective cohort study from the Taiwan National Health Insurance Research database reported that DOACs (including edoxaban) were associated with lower risks of ischemic stroke and major bleeding compared with warfarin [20]. These results were consistent across high-risk groups such as the elderly, those with chronic kidney disease, and those with a CHA_2_DS_2_-VASc score ≥4 [20]. In regard to representativeness across high-risk groups, one strength of the ETNA-AF study is the low proportion of patients that have missing CrCl data (11.6%). For comparison, the Xarelto for Prevention of Stroke in Patients with Atrial Fibrillation in Asia (XANAP) registry had missing CrCl data for almost half of its patients (48.5%) [21].

Adherence to treatment and prescription guidelines is an ongoing issue in Asian countries [22,23,24,25]. In a retrospective study of elderly Chinese patients with AF, only 44.7% of patients with a guideline-directed indication were treated with an oral anticoagulant [25]. DOACs have label-approved dose-reduction criteria; however, inappropriate under- or overdosing may influence clinical outcomes. In observational studies of Korean and Taiwanese patients with AF, only 61.9% and 68.9% of patients, respectively, were receiving the correct DOAC dose according to the label, and higher proportions of patients were underdosed vs. overdosed [22,23]. Underdosing was associated with a higher risk of thromboembolic events, while overdosing was associated with a higher risk of major bleeding [23]. The dosing stratification for the data used in the current analysis was published previously [26]. Overall, 70.8% of patients were prescribed the recommended edoxaban dose according to the local label [26]. Similar to previous studies, patients in the current analysis were more likely to receive the reduced dose of edoxaban (30 mg) without meeting the correct dose-reduction criteria according to the label (19.1% underdosed vs. 10.2% overdosed) [26]. However, this underdosing did not appear to impact clinical event rates (Supplementary Table S2). While off-label dosing can influence event rates, physicians may have clinical justification for not using the label-approved dose. For example, some patients may benefit from oral anticoagulation but may not meet eligibility criteria for the approved doses. A recent randomized placebo-controlled study demonstrated that a very low dose of edoxaban (15 mg) was beneficial in reducing the risk of stroke in very elderly Japanese patients (≥80 years of age) with AF who were not eligible for standard oral anticoagulation [27]. Additionally, in very elderly Chinese patients (≥85 years) with AF, the use of oral anticoagulation was associated with improved survival [28].

Some limitations of this prospective, noninterventional study include the lack of a comparator DOAC or VKA. Additionally, a longer duration of patient follow up and inclusion of more patients from other East Asian countries would provide further information for edoxaban use in routine clinical practice in the region. To that end, ETNA-AF has begun enrolling patients from additional Asian countries and/or regions, including mainland China, Hong Kong, and Thailand.

## 5. Conclusions

This analysis of Global ETNA-AF for patients receiving edoxaban from Korea and Taiwan showed a very low rate of clinical events at 1-year follow up and is further reassuring concerning the safety and effectiveness of edoxaban in routine clinical practice.

## Figures and Tables

**Table 1:** Key baseline demographics, clinical characteristics, and previous medical history.

	Total Patients (*n* = 2677)	Korea (*n* = 1769)	Taiwan (*n* = 908)
Age, median (IQR)	72 (66, 78)	72 (66, 77)	73 (67, 81)
≥75 years, *n* (%)	1083 (40.5)	668 (37.8)	415 (45.7)
Gender, male, *n* (%)	1597 (59.7)	1068 (60.4)	529 (58.3)
Weight, kg, median (IQR)	65 (57, 73)	65 (58, 73)	66 (57, 75)
<60 kg, *n* (%) ^a^	734 (29.5)	470 (29.4)	264 (29.7)
BMI, kg/m^2^, mean ± SD	25.0 ± 3.7	24.8 ± 3.5	25.4 ± 4.0
CHA_2_DS_2_-VASc, mean ± SD	3.1 ± 1.4	3.1 ± 1.4	3.2 ± 1.4
<2, *n* (%)	300 (11.2)	202 (11.4)	98 (10.8)
≥2, *n* (%)	2377 (88.8)	1567 (88.6)	810 (89.2)
HAS-BLED ^b^, mean ± SD	2.2 ± 1.0	2.1 ± 1.0	2.3 ± 1.1
CrCl, mL/min, median (IQR) ^c^	60.6 (47.1, 76.9)	62.6 (49.5, 79.1)	56.9 (41.7, 72.4)
<50 mL/min	706 (29.8)	390 (26.0)	316 (36.4)
≥50 mL/min	1661 (70.2)	1110 (74.0)	551 (63.6)
Type of AF, *n* (%)			
Paroxysmal	1163 (43.6)	608 (34.4)	555 (61.6)
Persistent	617 (23.1)	500 (28.3)	117 (13.0)
Long-standing persistent	390 (14.6)	306 (17.3)	84 (9.3)
Permanent	500 (18.7)	355 (20.1)	145 (16.1)
Edoxaban, *n* (%)			
60 mg	1304 (48.7)	909 (51.4)	395 (43.5)
On-label	1033 (38.6)	743 (42.0)	290 (31.9)
Off-label ^d^	271 (10.1)	166 (9.4)	105 (11.6)
30 mg	1373 (51.3)	860 (48.6)	513 (56.5)
On-label	863 (32.2)	513 (29.0)	350 (38.5)
Off-label ^e^	510 (19.1)	347 (19.6)	163 (18.0)
Previous medical history, *n* (%)			
Hypertension	1910 (71.3)	1257 (71.1)	653 (71.9)
Heart failure	324 (12.1)	177 (10.0)	147 (16.2)
Diabetes mellitus	788 (29.4)	503 (28.4)	285 (31.4)
Coronary artery disease	412 (15.4)	230 (13.0)	182 (20.0)
Peripheral arterial disease	21 (0.8)	6 (0.3)	15 (1.7)
COPD	132 (4.9)	70 (4.0)	62 (6.8)
Ischemic stroke	389 (14.5)	314 (17.8)	75 (8.3)
TIA	52 (1.9)	33 (1.9)	19 (2.1)
Major bleeding	56 (2.1)	36 (2.0)	20 (2.2)
ICH	38 (1.4)	29 (1.6)	9 (1.0)
Major GI bleeding	11 (0.4)	3 (0.2)	8 (0.9)
CRNM bleeding	22 (0.8)	10 (0.6)	12 (1.3)
Previous and concomitant medications, *n* (%) ^f^			
Antiarrhythmic/rate control drugs	1127 (42.1)	583 (33.0)	544 (59.9)
Antiplatelets	173 (6.5)	133 (7.5)	40 (4.4)

^a^ Missing: 170 from Korea and 20 from Taiwan. ^b^ Modified HAS-BLED: without “labile INR.” ^c^ Calculated using the Cockcroft–Gault equation; Missing: 269 from Korea and 41 from Taiwan. ^d^ Patients who met dose-reduction criteria but still received the standard 60 mg dose. ^e^ Patients who did not meet dose-reduction criteria but still received the reduced 30 mg dose. ^f^ Treatment was started on or before the date of the baseline visit and was continued after the baseline visit. AF, atrial fibrillation; BMI, body mass index; CHA_2_DS_2_-VASc, congestive heart failure, hypertension, age, diabetes, Prior Stroke/TIA, vascular disease, and sex category; COPD, chronic obstructive pulmonary disease; CrCl, creatinine clearance; CRNM, clinically relevant non-major; GI, gastrointestinal; HAS-BLED, Hypertension, abnormal renal and liver function, stroke, bleeding history or disposition, labile INR, elderly, drugs or alcohol; ICH, intracranial hemorrhage; INR, international normalized ratio; IQR, interquartile range; SD, standard deviation; TIA, transient ischemic attack.

**Table 2:** One-year clinical events.

	Total Patients (*n* = 2677)		Korea (*n* = 1769)		Taiwan (*n* = 908)	
	Events	%/Year (95% CI)	Events	%/Year (95% CI)	Events	%/Year (95% CI)
Major bleeding	20	0.78 (0.48–1.21)	8	0.47 (0.20–0.93)	12	1.40 (0.73–2.45)
ICH	7	0.27 (0.11–0.56)	3	0.18 (0.04–0.51)	4	0.47 (0.13–1.19)
Major GI bleeding	6	0.23 (0.09–0.51)	2	0.12 (0.01–0.42)	4	0.47 (0.13–1.19)
CRNM bleeding	12	0.47 (0.24–0.82)	3	0.18 (0.04–0.51)	9	1.05 (0.48–2.00)
Ischemic stroke	23	0.90 (0.57–1.35)	12	0.71 (0.36–1.23)	11	1.29 (0.64–2.30)
TIA	5	0.19 (0.06–0.46)	1	0.06 (0.00–0.33)	4	0.47 (0.13–1.20)
Hemorrhagic stroke	5	0.19 (0.06–0.45)	3	0.18 (0.04–0.51)	2	0.23 (0.03–0.84)
MI	4	0.16 (0.04–0.40)	2	0.12 (0.01–0.42)	2	0.23 (0.03–0.84)
SEE	1	0.04 (0.00–0.22)	1	0.06 (0.00–0.33)	0	0.00 (0.00–0.43)
All-cause mortality	30	1.17 (0.79–1.67)	13	0.76 (0.41–1.30)	17	1.98 (1.15–3.16)
CV mortality	13	0.51 (0.27–0.87)	8	0.47 (0.20–0.92)	5	0.58 (0.19–1.36)

CI, confidence interval; CRNM, clinically relevant non-major; CV, cardiovascular; GI, gastrointestinal; ICH, intracranial hemorrhage; MI, myocardial infarction; SEE, systemic embolic event; TIA, transient ischemic attack.

**Table 3:** Comparison of ETNA-AF Korea/Taiwan and ETNA-AF global one-year clinical events.

	Korea (*n* = 1769)	Taiwan (*n* = 908)	Global [13] (*n* = 26,823)
Major bleeding	0.47	1.40	1.12
ICH	0.18	0.47	0.31
Major gastrointestinal bleeding	0.12	0.47	0.57
Any stroke	0.88	1.52	1.12
Ischemic stroke	0.71	1.29	0.87
All-cause mortality	0.76	1.98	3.03
CV mortality	0.47	0.58	1.22

CV, cardiovascular; ETNA-AF, Edoxaban in rouTine cliNical prActice-atrial fibrillation; ICH, intracranial hemorrhage.

## Data Availability

Data from this analysis will not be made available because the study is still ongoing.

## Supplementary Materials

The following are available online at https://www.mdpi.com/article/10.3390/jcm10225337/s1, Table S1: One-year clinical events by edoxaban dose, Table S2: One-year clinical events by label-recommended edoxaban dose.
