# Supplementary material for: Clinical Events with Edoxaban in South Korean and Taiwanese Atrial Fibrillation Patients in Routine Clinical Practice
Source: J Clin Med. 2021 Nov 16;10(22):5337. doi: 10.3390/jcm10225337 (PMC8623093; doi:10.3390/jcm10225337)
Supplement: Supplementary file 1 [file jcm-10-05337-s001.zip › jcm-1411630-supplementary.pdf]

Table S1. One-year clinical events by edoxaban dose

	Total patients (N = 2677)		Korea (n = 1769)		Taiwan (n = 908)	
	EDX 30 mg n = 1373	EDX 60 mg n = 1304	EDX 30 mg n = 860	EDX 60 mg n = 909	EDX 30 mg n = 513	EDX 60 mg n = 395
Major bleeding	17 (1.31)	3 (0.24)	6 (0.73)	2 (0.23)	11 (2.31)	1 (0.26)
ICH	7 (0.53)	0	3 (0.36)	0	4 (0.83)	0
Major GI bleeding	6 (0.46)	0	2 (0.24)	0	4 (0.83)	0
CRNM bleeding	6 (0.46)	6 (0.48)	2 (0.24)	1 (0.11)	4 (0.84)	5 (1.33)
Ischemic stroke	12 (0.92)	11 (0.88)	4 (0.48)	8 (0.92)	8 (1.67)	3 (0.80)
TIA	4 (0.31)	1 (0.08)	1 (0.12)	0	3 (0.63)	1 (0.26)
Hemorrhagic stroke	5 (0.38)	0	3 (0.36)	0	2 (0.42)	0
MI	3 (0.23)	1 (0.08)	2 (0.24)	0	1 (0.21)	1 (0.26)
SEE	0	1 (0.08)	0	1 (0.11)	0	0
All-cause mortality	24 (1.83)	6 (0.48)	9 (1.08)	4 (0.46)	15 (3.11)	2 (0.53)
CV mortality	10 (0.76)	3 (0.24)	5 (0.60)	3 (0.34)	5 (1.04)	0

All data presented as *n* (%/year). CRNM, clinically relevant non-major; CV, cardiovascular; EDX, edoxaban; GI, gastrointestinal; ICH, intracranial hemorrhage; MI, myocardial infarction; SEE, systemic embolic event; TIA, transient ischemic attack.

Table S2. One-year clinical events by label-recommended edoxaban dose

	**Total patients** *N* **= 2677**			
	On-label EDX 30 mg *n* = 863 (32.2%)	Off-label EDX 30 mg *n* = 510 (19.1%)	On-label EDX 60 mg *n* = 1033 (38.6%)	Off-label EDX 60 mg *n* = 271 (10.1%)
Major bleeding	13 (1.60)	4 (0.82)	3 (0.30)	0
ICH	6 (0.74)	1 (0.20)	0	0
Major GI bleeding	5 (0.61)	1 (0.20)	0	0
CRNM bleeding	5 (0.61)	1 (0.20)	5 (0.50)	1 (0.38)
Ischemic stroke	8 (0.98)	4 (0.81)	9 (0.91)	2 (0.77)
TIA	4 (0.49)	0	0	1 (0.39)
Hemorrhagic stroke	4 (0.49)	1 (0.20)	0	0
MI	1 (0.12)	2 (0.41)	0	1 (0.39)
SEE	0	0	1 (0.10)	0
All-cause mortality	20 (2.44)	4 (0.81)	4 (0.40)	2 (0.77)
CV mortality	8 (0.98)	2 (0.41)	3 (0.30)	0

All data presented as *n* (%/year). Off-label refers to patients receiving the 30-mg dose without meeting dose reduction criteria or patients who do meet dose reduction criteria receiving the standard 60-mg dose. These groups were referred to as “recommended” and “non-recommended” dosing in a previously published report [26].

CRNM, clinically relevant non-major; CV, cardiovascular; EDX, edoxaban; GI, gastrointestinal; ICH, intracranial hemorrhage; MI, myocardial infarction; SEE, systemic embolic event; TIA, transient ischemic attack.
